# Improving the usefulness of evidence concerning the effectiveness of implementation strategies for knowledge products in primary healthcare: protocol for a series of systematic reviews

**DOI:** 10.1186/s13643-020-01382-x

**Published:** 2020-05-19

**Authors:** Hervé Tchala Vignon Zomahoun, José Massougbodji, André Bussières, Aliki Thomas, Dahlia Kairy, Claude Bernard Uwizeye, Nathalie Rheault, Ali Ben Charif, Ella Diendéré, Léa Langlois, Sébastien Tchoubi, Serigne Abib Gaye, France Légaré

**Affiliations:** 1 Health and Social Services Systems, Knowledge Translation and Implementation Component of the Quebec SPOR SUPPORT Unit, Laval University, Quebec, Quebec, Canada; 2 Centre intégré universitaire de santé et de services sociaux de la Capitale-Nationale (CIUSSS-CN), Quebec, Québec Canada; 3 Centre de recherche sur les soins et les services de première ligne de l’Université Laval (CERSSPL-UL), Pavillon Landry-Poulin – 2525, Chemin de la Canardière, Québec, G1J 0A4 Canada; 4 Department of Social and Preventive Medicine, Laval University, Quebec, Quebec Canada; 5 School of Physical and Occupational Therapy, Faculty of Medicine, McGill University, Montreal, Quebec, Canada; 6 Centre de recherche interdisciplinaire en réadaptation du Montréal métropolitain (CRIR), Montreal, Quebec, Canada; 7 Réseau provincial de recherche en adaptation-réadaptation (REPAR), Montreal, Quebec, Canada; 8 Département chiropratique, Université du Québec à Trois-Rivières, Trois-Rivières, Quebec, Canada; 9 School of Rehabilitation, Université de Montréal, Montreal, Quebec, Canada; 10 Canada Research Chair in Shared Decision Making and Knowledge Translation, Laval University, Quebec, Quebec Canada; 11 Department of Family Medicine and Emergency Medicine, Laval University, Quebec, Quebec Canada

**Keywords:** Implementation strategies, Primary healthcare, Overviews, Systematic reviews, Meta-analysis

## Abstract

**Background:**

The literature on the implementation of knowledge products is extensive. However, this literature is still difficult to interpret for policymakers and other stakeholders when faced with choosing implementation strategies likely to bring about successful change in their health systems. This work has the particularity to examine the scope of this literature, and to clarify the effectiveness of implementation strategies for different knowledge products. Consequently, we aim to (1) determine the strengths and weaknesses of existing literature overviews; (2) produce a detailed portrait of the literature on implementation strategies for various knowledge products; and (3) assess the effectiveness of implementation strategies for each knowledge product identified and classify them.

**Methods:**

We will use a three-phase approach consisting of a critical analysis of existing literature overviews, a systematic review of systematic reviews, and a series of systematic reviews and meta-analyses. We will follow the Cochrane Methodology for each of the three phases. Our eligibility criteria are defined following a PICOS approach: *Population*, individuals or stakeholders participating in healthcare delivery, specifically, healthcare providers, caregivers, and end users; *Intervention*, any type of strategy aiming to implement a knowledge product including, but not limited to, a decision support tool, a clinical practice guideline, a policy brief, or a decision-making tool, a one-pager, or a health intervention; *Comparison*, any comparator will be considered; *Outcomes*, *phases 1 and 2*—any outcome related to implementation strategies including, but not limited to, the measures of adherence/fidelity to the use of knowledge products, their acceptability, adoption, appropriateness, feasibility, adaptability, implementation costs, penetration/reach and sustainability; *phase 3*—any additional outcome related to patients (psychosocial, health behavioral, and clinical outcomes) or healthcare professionals (behavioral and performance outcomes); *Setting*, primary healthcare has to be covered. We will search MEDLINE (Ovid), EMBASE, Web of Science, PsycINFO, CINAHL, and the Cochrane Library from their inception onwards. For each phase, two reviewers will independently perform the selection of studies, data extraction, and assess their methodological quality. We will analyze extracted data, and perform narrative syntheses, and meta-analyses when possible.

**Discussion:**

Our results could inform not only the overviews’ methodology but also the development of an online platform for the implementation strategies of knowledge products. This platform could be useful for stakeholders in implementation science.

**Systematic review registration:**

Protocol registered on Open Science Framework, https://osf.io/eb8w2/.

## Background

### Description of the condition

Having an evidence-based knowledge product does not mean that it will be systematically implemented with success and be beneficial for patients. Balas et al. [1] estimated that it takes up to 17 years to translate 14% of original research into practices for the benefit of patients. Given this gap between the production of health evidence and its application in routine practice, increasing attention has been devoted to knowledge translation (KT), and more specifically, to KT [2, 3] and implementation strategies [3–5]. Knowledge products considered in implementation strategies include clinical practice guidelines, shared decision-making tools, decision support tools, policy briefs, a one-pager (simple, iconographic, infographic), and a health intervention (technological, pharmacological, behavioral, or management). As a result, there has been a substantial increase in studies examining their effectiveness.

### Description of the implementation

Implementation can be defined as an actively planned and deliberatively initiated effort with the intention to translate a given knowledge product into practices in a particular setting and context [6, 7]. Implementation requires characterizing the knowledge product to be implemented as well as the context and setting of implementation [7]. It is also important to (1) determine an adapted process framework or theory; (2) clarify the different stages of the implementation process; (3) identify stakeholders to be involved; (4) recognize facilitators and barriers to implementation using an appropriate framework; (5) identify the corresponding implementation strategies; and (6) target the relevant outcomes and tools to evaluate them [7]. Implementation strategies have been explored extensively in the literature. They can be defined as techniques or methods aiming to improve or optimize the uptake and routine application of complex interventions in clinical care [5]. To be better characterized, implementation strategies have to be named, defined, and specified [5]. The Cochrane Effective Practice and Organization of Care (EPOC) grouped existing implementation strategies in categories targeting respectively healthcare organizations, healthcare professionals, specific types of practice, condition, or setting [3].

### How the knowledge product implementation might work

The implementation of a given knowledge product should be guided by one or more frameworks/models/theories depending on its complexity. As suggested by Nilsen [8], an implementation process, identification of its determinants, and evaluation of its outcomes may be supported by a framework/model/theory. Indeed, frameworks/models/theories permit stakeholders to better explain and clarify different steps of the implementation process, i.e., a description of the main steps to translate a given knowledge product into practices followed by the evaluation of outcomes and the sustainability of its use (e.g., the knowledge to action framework [9]). Frameworks/models/theories can also be used to better address the various levels of factors that potentially influence implementation outcomes (e.g., the Consolidated Framework for Implementation Research, CFIR [7]). Finally, there are frameworks/models/theories that have been developed to optimally address the various levels of outcomes that can both explain how different steps of the implementation process took place as well as indicate the timeline for their evaluation [10, 11].

### Why it is important to do a series of systematic reviews

According to our preliminary literature search, we found that the literature on the implementation of knowledge products is large. In an effort to further synthesize, several overviews of these systematic reviews [4, 12–21] have been published in the last 20 years. Overviews attempt to systematically retrieve and summarize the results of multiple systematic reviews on the effectiveness of interventions or treatments for a given condition or public health problem [22]. Their purpose is to contrast the findings of several systematic reviews on the same subject, thus offering decision-makers a broader understanding of the evidence. While, in theory, this type of study has the potential to overview the evidence, its execution is fraught with several challenges. There are guidelines for conducting this type of research [23–26]; however, several experts who have examined the methodology regarding overviews of systematic reviews over the past decade agreed that existing directives and recommendations are not sufficiently robust and there is great room for improvement [27–29].

This literature remains difficult to interpret for policymakers and other stakeholders when faced with having to select implementation strategies likely to bring about successful change in their health systems. Indeed, it remains a challenge to determine what strategy is effective for the implementation of a given knowledge product. Examining the scope of this literature and further clarifying the effectiveness of implementation strategies for different knowledge products could serve to identify original and relevant research questions for future systematic reviews. In turn, these latter may allow us to quantitatively assess the effectiveness of implementation strategies for specific knowledge products. The general aim of this project is to identify, for each category of knowledge product, the most effective implementation strategies for their uptake into healthcare professionals’ clinical practice.

We aim to address the following specific objectives in three distinct phases:

1. Phase 1—to perform a critical analysis of existing literature overviews to determine their strengths and weaknesses. This analysis will not only help us better define our research question for subsequent phases but will also highlight many of the methodological challenges we may encounter.
2. Phase 2—to conduct a systematic review of systematic reviews while taking into account the weaknesses of previously published overviews, and provide a detailed portrait of the literature on various implementation strategies for knowledge.
3. Phase 3—to perform a series of meta-analyses that evaluate the effectiveness of implementation strategies for the different types of knowledge products, using individual randomized controlled trials (RCTs) from systematic reviews included in phase 2. These analyses will provide answers to specific research questions with the most robust experimental study designs, and more detailed ones, in which contextual elements, often absent in overviews, are going to be taken into consideration.

## Methods

This study protocol has been registered within the Open Science Framework platform (Registration ID: https://osf.io/eb8w2/). We are reporting it in accordance with the reporting guidance provided in the Preferred Reporting Items for Systematic Reviews and Meta-Analyses Protocols (PRISMA-P) statement (See PRISMA-P checklist in Additional file 2) [30]. We will conduct this project following the recommendations in the Cochrane Handbook for systematic reviews [31].

**Fig. 1:**
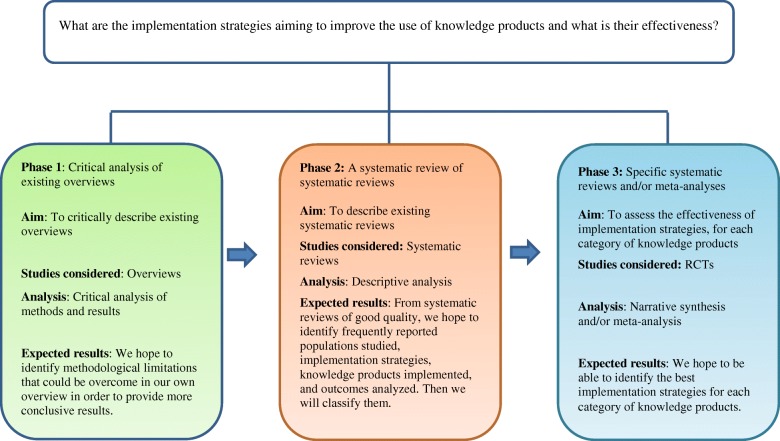
Three-phase approach used for the present review

We will use a three-phase approach (see Fig. 1 for details) including a critical analysis of existing literature overviews, a systematic review of systematic reviews, and a series of systematic reviews and meta-analyses.

### Eligibility criteria

In the first two phases of the project, according to a PICOS (**P**opulation, **I**ntervention, **C**omparison, **O**utcomes, **S**tudy design) with knowledge products, studies that meet the following criteria will be considered for inclusion:

#### Population

The population of interest is individuals or stakeholders participating in healthcare delivery, specifically, healthcare providers, caregivers, and end users (i.e., patients or clients with any conditions).

#### Intervention

We will consider any type of strategy aiming to implement a knowledge product. The EPOC taxonomy will be used to classify implementation strategies identified (e.g., audit and feedback, educational meetings, interprofessional education) [3].

#### Knowledge product

As suggested by Pinnock et al. [32], a clear distinction has been made between the implementation strategies and the knowledge products implemented. A knowledge product could be, but not limited to, a decision support tool, a clinical practice guideline, a policy brief, or a decision-making tool, a one-pager (simple, iconographic, infographic), or a health intervention (technological, pharmacological, behavioral, or management). Only implementation studies in which both are reported will be considered in our project.

#### Comparison

Any comparator will be considered. This includes studies in which two or more implementation strategies for knowledge products are compared (head to head); and studies in which there is no comparator.

#### Outcome(s)

In phases 1 and 2, we will mainly consider outcomes related to implementation strategies including, but not limited to, the measures of adherence/fidelity to the use of knowledge products, their acceptability, adoption, appropriateness, feasibility, adaptability, implementation costs, penetration/reach, and sustainability [11]. As for phase 3, we will additionally consider the outcomes related to patients (psychosocial, health behavioral, and clinical outcomes) or healthcare professionals (psychosocial, health behavioral, and performance outcomes) that the knowledge products aim to improve.

#### Study design

This eligibility criterion will be specific to each phase of the project:

- *Phase 1*. We will consider any review of reviews on implementation strategies for knowledge products (i.e., a knowledge synthesis in which included studies are any literature reviews).
- *Phase 2*. We will consider only systematic reviews (i.e., any study in which authors performed a narrative and/or quantitative synthesis of experimental studies on implementation strategies for knowledge products using a comprehensive and reproducible approach).
- *Phase 3*. Only RCTs will be considered.

#### Setting

Any health domain addressed in primary healthcare will be considered. A review will be included if primary healthcare is covered.

### Information sources and search strategy

We will perform comprehensive searches within five electronic databases: MEDLINE (Ovid), EMBASE, Web of Science, PsycINFO, CINAHL, and the Cochrane Library from their inception onwards. The search strategy will be designed by an information specialist using “knowledge transfer” and “implementation strategies” headings (See draft search strategy for MEDLINE/Ovid in Additional file 1). For efficiency considerations, we will not build a new search strategy to identify RCTs, rather, we plan in identifying them from the systematic reviews that we will select in our overview. However, to avoid missing any recently published RCTs in the field, we will update our search strategy by looking for primary studies published after the most recent included reviews. Searches will be conducted within the same electronic databases as in earlier phase. Study designs other than RCTs, for example systematic reviews, quasi-experimental studies and observational studies, will be excluded.

There will be neither language nor literature search date restrictions. The references listed in all eligible studies will also be manually searched in order to identify additional relevant ones.

### Data management

We will merge the citations identified from five electronic databases mentioned above in the EndNote software. Then, we will identify and remove the duplicates. The unique citations will be considered for the selection process.

### Selection of studies

Two assessors will independently contribute to all steps of the selection process. References identified from relevant electronic databases will be merged, and duplicates removed to obtain a database including unique citations for the study selection process. Assessors will discuss the inclusion criteria to ensure mutual understanding, and pilot test on five percent of unique citations identified to confirm that the evaluation process is reliable. The pilot section will be considered conclusive if the kappa statistic referring to the agreement between assessors is greater than 0.7 [33].

Pairs of assessors will then independently screen for titles and abstracts based on inclusion criteria specific to each phase outlined above. In case of doubt, the citations will be included and considered for full-text reading. Assessors will then independently screen the full texts of references retained at the first step. Any disagreement will be resolved by consensus or by involving a third assessor.

Assessors will include overviews for phase 1 and systematic reviews for phase 2, based on the eligibility criteria mentioned above. For phase 3, the following procedure will be adopted for the identification of relevant RCTs from each review considered: first, we will go through the list including primary studies to identify RCTs that are clearly reported as such by the review’s authors. In the case, RCTs are not, we will select the complete list of primary articles included. Removal of duplicates and screening based only on the study design will be done by two independent assessors.

### Data extraction

For each phase, a pilot data extraction will be done on 5% of selected articles. The data extraction will be pursued only when the pilot is conclusive. This process will be independently performed by two assessors. Discrepancies between them will be resolved by a third author.

#### Phase 1

The following data will be extracted: the first author’s name; the year of publication; the focus of the overview, based on the scope of PICO elements; the number and name of databases consulted; the period of the literature search; the strategies used to update the literature; the type and number of reviews that are included; the type of reviews (Cochrane, non-Cochrane, both); the strategies to deal with the overlap between reviews; the strategies to deal with conflicting results of reviews; the quality assessment of reviews; the type of synthesis performed (narrative, meta-analysis or both); the assessment of the quality of evidence, and the limitations reported by overview authors.

#### Phase 2

We will extract data concerning the following elements:

- *Review details*. First author’s name, year of publication, type, objectives, registration information.
- *Literature search details*. Number and names of electronic databases, search for gray literature, search period, restrictions, design and number of primary studies.
- *Characteristics of participants*. Name, profile, total number, age (mean, median, and/or range), gender (percentage of men or women).
- *Characteristics of implementation strategies*. Components described according to the EPOC taxonomy, knowledge products implemented (e.g., clinical guidelines, health intervention).
- *Characteristics of outcomes*. Name, type of measurement tools used (self-administered, objective, both). We will use Proctor et al. taxonomy to guide the classification of outcomes [11].
- *Assessment of study quality*. Name of the tool used, result.
- *Type of synthesis*. Narrative, meta-analysis, or both.
- *Assessment of publication bias*. Method used (funnel plots, statistical tests, both), and treatment of it if present.
- *Assessment of the quality of evidence*. Name of the tool used, level of evidence.
- *Conclusion of reviews*. Information reported by authors when they conclude (e.g., main results and limitations).

#### Phase 3

For each RCT considered in this phase, we will extract data concerning the following elements:

- *Characteristics of the study*. First author’s name, year of publication, country, language, healthcare domain, setting, study design, sampling, and recruitment method.
- *Characteristics of participants*. Size of eligible population for both healthcare providers and end users, total sample size, sample sizes in implementation strategy, and control groups, unit of allocation, mean age or range, sex, race/ethnicity (percentage of Caucasians), socioeconomic level of the sampled population, and profile.
- *Characteristics of implementation strategies (description of the intervention)*. Components (according to the EPOC taxonomy), underlying framework, content, number of participating centers, mode of delivery (e.g., in person and online), actual duration (e.g., number of hours), actual frequency (e.g., number of sessions), methods used (e.g., interactive or didactic techniques, web applications), course materials used (e.g., web capsules, textbooks), adherence/fidelity, and content of the intervention offered to control group.
- *Characteristics of knowledge products implemented*. Name, format and content.
- *Characteristics of outcomes and analysis methods used*. Names of interest outcomes, measurement tools, range of scale and analysis methods used, unit of analysis, intention to treat analysis or not, retention rates, and effect measures of implementation strategies.
- *Effects of implementation strategies*. Any information useful to estimate the effect size and its 95% confidence interval will be extracted. For continuous outcomes, we will consider the standardized mean difference as effect size requiring the sample size, mean score, and standard deviation in each group studied. For dichotomous outcomes, we will consider the odds ratio as effect size requiring the sample size and number of events in each group studied. We will also collect the value of effect size (crude and adjusted) and its confidence interval estimated in the studies. Information about confounding factors will as well be extracted.

When necessary, corresponding or first authors will be contacted to obtain information about missing data in their studies.

### Quality assessment of studies

The quality of overviews retrieved in phase 1 will not be assessed since there is no methodological tool designed for this purpose. Moreover, the objective of this phase is not to draw any conclusions on the effectiveness of implementation strategies from these overviews.

For phase 2, we will assess the methodological quality of selected systematic reviews using the AMSTAR 2 tool [34]. Those meeting the criteria of good or medium quality will be retained for the rest of the process, i.e., will be considered for the next steps of the review of reviews and for the identification of RCTs in phase 3.

As for phase 3, RCTs extracted from systematic reviews will be assessed with the Cochrane Risk of Bias (RoB) 2.0 tool [35].

Both the quality assessment of systematic reviews as the assessing risk of bias for RCTs will be performed independently by two authors. In a pilot assessment of two or four studies included, they will agree on a common understanding of the definitions, criteria, and guidelines provided in the tool to achieve a more objective assessment. The assessment of all studies included will do once the pilot test is conclusive. When needed, a third reviewer will be invited to help reach consensus.

### Data synthesis

#### Phase 1

We will perform a descriptive analysis using counts (number and percentage) to summarize data collected followed by carrying out a critical analysis on the latter considering the following methodological elements: literature search, methodological limitations reported in overviews, overlap between reviews included in overviews, types of data synthesis, quality of reviews included in overviews, and level of evidence.

#### Phase 2

We will produce a descriptive Preferred Reporting Items for Systematic Reviews and Meta-Analyses (PRISMA) flowchart of the selection process for all included reviews [36]. We will then describe the characteristics of the latter, populations, implementation strategies, and outcomes assessed. We will also analyze the data on publication bias and quality of evidence assessment. Finally, evaluation data of included reviews from the AMSTAR 2 tool [34] will be presented using a graph and table.

#### Phase 3

First, we will classify RCTs according to the type of knowledge product being implemented. These may include but not limited to clinical guidelines, decision support tools, research summaries, one-pagers (simple, iconographic, infographic), or other health interventions (technological, pharmacological, behavioral, or management).

Second, we will present a descriptive flowchart of the RCTs included in each meta-analysis, according to the PRISMA guidelines [36]. We will describe by frequencies (number and percentage) the characteristics of studies, populations, implementation strategies, and outcomes. Risk of bias levels will also be described for all included RCTs.

Finally, for each outcome and knowledge product, we will determine if there are sufficient data to perform a meta-analysis. If not, we will conduct a narrative synthesis on the effects of implementation strategies, and include in tables and figures to aid in data presentation. If so, we will do a meta-analysis. For each outcome and knowledge product, the random effect model will be used to estimate the pooled effect size of an implementation strategy and its 95% confidence interval, as we anticipate heterogeneity among the RCTs concerning the types of implementation intervention and population [31, 37]. For dichotomous data, the effect size will be expressed as a risk ratio or odds ratio; for continuous data, it will be provided as a standardized mean difference if different measures are used for the same outcome. For cluster RCTs, the analysis will be adjusted for clustering to avoid unit-of-analysis errors [31]. The influence of clinical and methodological heterogeneity on the observed effects will be discussed. Subgroup analyses will be carried out if necessary according to characteristics of the studies, the populations and the implementation interventions mentioned above. Statistical heterogeneity will be assessed using the Higgins’ I square statistic [38, 39]. A funnel plot will be generated to assess publication bias if 10 or more studies are included in the meta-analysis [40]. Statistical tests for funnel plot asymmetry (e.g., Egger’s regression, Begg’s test, and Harbord’s test) will be performed where appropriate [31, 40].

Sensitivity analyses will be carried out excluding the RCTs with high risk of bias from pooled effect size estimates. For each outcome and knowledge product, we will also explore the influence of each RCT by removing its individual effect size from the pooled estimation. These different analyses will allow us to evaluate the robustness of our results.

### Assessment of the quality of evidence

The quality of evidence for each outcome and knowledge product will be assessed with the Grading of Recommendations, Assessment, Development and Evaluation (GRADE) to reduce the misinterpretation of our review’s results [41]. The GRADE tool is based on five criteria for each individual study, namely risk of bias, indirectness of evidence, data heterogeneity, imprecision of effect size estimates, and risk of publication bias [41]. Overall assessment will be rated very low, low, moderate, and high for each outcome [41].

## Discussion

Our project aims to improve the usefulness of evidence on implementation strategies for knowledge products in primary healthcare. Our expected findings will have both methodological and practice implications. First, findings from the present project, mainly the ones related to the critical analysis on methodology, will contribute to methodological aspects for designing and conducting literature overviews. To this end, we will identify methodological limitations in overviews, and formulate corresponding recommendations with our collaborative team of methodologists. These recommendations will be applied to our own overview in order to determine their applicability. Second, our findings will also serve to provide a list of implementation strategies for various knowledge products. This list will include established effective implementation strategies, those potentially effective, and the non-effective ones for each knowledge product identified. These findings will inform the development of an online platform on implementation strategies for knowledge products in primary healthcare.

Apart from the methodological challenges already taken into account in our methods section, we anticipate other limitations in the realization of our project. First, there may be discrepancies between what we have planned in this protocol and what we will finally do. So, we plan for justifying them and documenting important protocol amendments. Second, doing a series of systematic reviews can take a long time, so it could be challenging to keep update the literature search of interest. To save time, we plan to create parallel review teams that will simultaneously work at the steps of study selection, data extraction, and/or study quality assessment for the critical analysis of existing overviews and the systematic reviews of systematic reviews. For each systematic review of RCTs on specific research questions, we plan to systematically update our literature search to avoid missing any recently published RCTs.

We will use passive and active strategies to disseminate our findings.

### Passive strategies

Findings from this project will be published in leading journals in the field intended for knowledge users and stakeholders in implementation science. We plan to produce a series of manuscripts for publication in peer-reviewed journals organized in the following:

- A manuscript as a preamble in which we will report results from our critical analysis of existing overviews
- A paper describing results from our overview and our original methodology
- At least four manuscripts corresponding to each specific meta-analysis or narrative synthesis we will perform. In these papers, we will identify effective implementation strategies for each type of knowledge product

The findings will also be presented at local, national, and international conferences (number equal to four or six). Specialized conferences whose themes related to knowledge translation in health, implementation science, or primary healthcare will be privileged (e.g., the Annual Scientific Meeting of Knowledge Translation Canada, the Annual Conference on the Science of Dissemination and Implementation in Health, or the Annual Meeting of North American Primary Care Research Group). In these cases, we will solicit our expert partners on knowledge translation to participate as credible messengers. Finally, we will design a database on our website (http://unitesoutiensrapqc.ca/) on the effectiveness of implementation strategies stratified according to the types of outcomes and knowledge products. This database will be useful for anyone who wants to design an implementation study on a knowledge product.

### Active strategies

Findings from this project are of critical relevance to each of the following knowledge platforms or teams: the Knowledge Translation Components of Strategy for Patient-Oriented Research (SPOR) - Support for People and Patient-Oriented and Trials (SUPPORT) Units, the *Réseau provincial de recherche en adaptation-réadaptation (*REPAR), and the *Centre de recherche interdisciplinaire en réadaptation* (CRIR). One of the mandates from these organizations is to guide the application of evidence-based knowledge in primary care clinical practices, as well as in rehabilitation. Since our findings will be the product of a systematic approach, we will integrate them into the range of services we offer, including training workshops (e.g., for conferences or as interactive online training with a follow-up) targeting specific audiences such as health administrators/decision-makers/health professionals and patients. We will also develop a platform on implementation strategies for knowledge products in primary healthcare. A video capsule in plain language will be created to support the use of this platform and to train the potential users including healthcare professionals, health system decision-makers, patients, and researchers involving in implementation science. We will use various communication channels to publicize our platform: social media (e.g., Facebook, Twitter), our bimonthly newsletter (more than 500 subscribers), and clients’ emails through the database, particularly those who accepted to be contacted for our advertisements.

## Supplementary information

**Additional file 1.** Detailed search strategy in Ovid Medline.

**Additional file 2.** PRISMA-P 2015 Checklist.

## Data Availability

All data and materials used at this step are available in our protocol. There are currently no additional data or materials. However, all data and materials used during the review will be available from the corresponding author.

## Abbreviations

AMSTAR: A MeaSurement Tool to *Assess systematic Reviews*
CFIR: Consolidated Framework for Implementation Research
CINHAL: Cumulative Index to Nursing and Allied Health Literature
CIRR: *Centre de recherche interdisciplinaire en réadaptation*
EMBASE: Excerpta Medica dataBASE
EPOC: Effective Practice and Organization of Care
GRADE: Grading of Recommendations, Assessment, Development and Evaluation
KT: Knowledge translation
MEDLINE: Medical Literature Analysis and Retrieval System Online
REPAR: *Réseau provincial de recherche en adaptation-réadaptation*
RCTs: Randomized controlled trials
PICO: Population, Intervention, Comparison, Outcomes
PRISMA: Preferred Reporting Items for Systematic Reviews and Meta-Analyses
PRISMA-P: Preferred Reporting Items for Systematic Reviews and Meta-Analyses Protocols
SPOR: Strategy for Patient-Oriented Research
SUPPORT: Support for People and Patient-Oriented and Trials
