# Supplementary material for: Improving the usefulness of evidence concerning the effectiveness of implementation strategies for knowledge products in primary healthcare: protocol for a series of systematic reviews
Source: Syst Rev. 2020 May 19;9:112. doi: 10.1186/s13643-020-01382-x (PMC7236932; doi:10.1186/s13643-020-01382-x)
Supplement: Supplementary file 1 — Additional file 1. Detailed search strategy in Ovid Medline. [file 13643_2020_1382_MOESM1_ESM.docx]

| **Additional file 1. Detailed search strategy in Ovid Medline** | | |
| --- | --- | --- |
| **Concepts** | **Keywords for search strategy** | **#** |
| Knowledge translation (Controlled vocabulary) | TRANSLATIONAL MEDICAL RESEARCH/ or Information Dissemination/ or exp "diffusion of innovation"/ or exp Education, Continuing/mt, st [Methods, Standards] | #1 |
| Strategies | (Strategy or strategies or tool* or framework* or intervention* or program or programs or programme*).ti. or (strategy or strategies or tool* or framework* or intervention* or program or programs or programme*).ab. or (strategy or strategies or tool* or framework* or intervention* or program or programs or programme* ).kf. | #2 |
| Knowledge translation strategies | 1 AND 2 | #3 |
| Knowledge translation (Free text) | (("knowledge to action" or "KT" or implementation or implementing or disseminat*) adj3 (strategy or strategies or tool* or framework* or intervention* or program or programs or programme*)).ti. or (("knowledge to action" or "KT" or implementation or implementing or disseminat*) adj3 (strategy or strategies or tool* or framework* or intervention* or program or programs or programme*)).ab. or (("knowledge to action" or "KT" or implementation or implementing or disseminat*) adj3 (strategy or strategies or tool* or framework* or intervention* or program or programs or programme*)).kf. | #4 |
|  | (knowledge adj3 (transfer* or translat* or broker* or uptake or "up take" or exchange* or application or utili#ation or cycle or transform* or action or diffusion) adj3 (strategy or strategies or tool* or framework* or intervention* or program or programs or programme*)).ti. or (knowledge adj3 (transfer* or translat* or broker* or uptake or "up take" or exchange* or application or utili#ation or cycle or transform* or action or diffusion) adj3 (strategy or strategies or tool* or framework* or intervention* or program or programs or programme*)).ab. or (knowledge adj3 (transfer* or translat* or broker* or uptake or "up take" or exchange* or application or utili#ation or cycle or transform* or action or diffusion) adj3 (strategy or strategies or tool* or framework* or intervention* or program or programs or programme*)).kf. | #5 |
|  | (translat* adj3 gap adj3 (strategy or strategies or tool* or framework* or intervention* or program or programs or programme*)).ti. or (translat* adj3 gap adj3 (strategy or strategies or tool* or framework* or intervention* or program or programs or programme*)).ab. or (translat* adj3 gap adj3 (strategy or strategies or tool* or framework* or intervention* or program or programs or programme*)).kf. | #6 |
|  | (research adj3 uptake adj3 (strategy or strategies or tool* or framework* or intervention* or program or programs or programme*)).ti. or (research adj3 uptake adj3 (strategy or strategies or tool* or framework* or intervention* or program or programs or programme*)).ab. or (research adj3 uptake adj3 (strategy or strategies or tool* or framework* or intervention* or program or programs or programme*)).kf. | #7 |
|  | (educational adj3 outreach adj3 (strategy or strategies or tool* or framework* or intervention* or program or programs or programme*)).ti. or (educational adj3 outreach adj3 (strategy or strategies or tool* or framework* or intervention* or program or programs or programme*)).ab. or (educational adj3 outreach adj3 (strategy or strategies or tool* or framework* or intervention* or program or programs or programme*)).kf. | #8 |
|  | ((opinion or education* or influential) adj2 (leader or leaders) adj3 (strategy or strategies or tool* or framework* or intervention* or program or programs or programme*)).ti. or ((opinion or education* or influential) adj2 (leader or leaders) adj3 (strategy or strategies or tool* or framework* or intervention* or program or programs or programme*)).ab. or ((opinion or education* or influential) adj2 (leader or leaders) adj3 (strategy or strategies or tool* or framework* or intervention* or program or programs or programme*)).kf. | #9 |
|  | (evidence* adj2 practice* adj3 (strategy or strategies or tool* or framework* or intervention* or program or programs or programme*)).ti. or (evidence* adj2 practice* adj3 (strategy or strategies or tool* or framework* or intervention* or program or programs or programme*)).ab. or (evidence* adj2 practice* adj3 (strategy or strategies or tool* or framework* or intervention* or program or programs or programme*) ).kf. | #10 |
|  | (education* adj1 (intervention* or strateg*)).ti. or (education* adj1 (intervention* or strateg*)).ab. or (education* adj1 (intervention* or strateg*)).kf. | #11 |
| Knowledge Translation (Free text) | ((evidence or research or knowledge) adj5 (bridge or close)).ti. or ((evidence or research or knowledge) adj5 (bridge or close)).ab. or ((evidence or research or knowledge) adj5 (bridge or close)).kf. | #12 |
| Strategies (Free text) | (strategy or strategies or tool* or framework* or intervention* or program or programs or programme*).ti. or (strategy or strategies or tool* or framework* or intervention* or program or programs or programme*).ab. or (strategy or strategies or tool* or framework* or intervention* or program or programs or programme*).kf. | #13 |
|  | 12 AND 13 | #14 |
| Knowledge translation strategies | 3 OR 4 OR 5 OR 6 OR 7 OR 8 OR 9 OR 10 OR 11 OR 14 | #15 |
| Filter for review (free text) | (bibliographic* or review? or meta-analy* or metaanaly* or overview* or ((research or literature) adj3 synthesis) or ((information or data or evidence*) adj3 synthesis) or (data adj2 extract*)).ti. or (bibliographic* or review or meta-analy* or metaanaly* or overview* or ((research or literature) adj3 synthesis) or ((information or data or evidence*) adj3 synthesis) or (data adj2 extract*)).ab. or (cinahl or (cochrane adj3 trial*) or embase or medline or psyclit or (psycinfo not "psycinfo database") or pubmed or scopus or "sociological abstracts" or "web of science").ab. or ("cochrane database of systematic reviews" or evidence report technology assessment or evidence report technology assessment summary ).jn. or (review or Meta-Analysis).pt. | #16 |
| Filter for review (Controlled vocabulary) | meta-analysis as topic/ or review literature as topic/ | #17 |
| Filter for review | 16 OR 17 | #18 |
| Health Professionals (Controlled vocabulary) | exp Professional-Patient Relations/ or exp Health Personnel/ | #19 |
| Health Professionals (Free text) | (nurse* or physician* or clinician* or doctor* or generalist* or practitioner* or provider* or professional* or personnel* or resident* or staff* or team or teams).ti. or (nurse* or physician* or clinician* or doctor* or generalist* or practitioner* or provider* or professional* or personnel* or resident* or staff* or team or teams).ab. or (nurse* or physician* or clinician* or doctor* or generalist* or practitioner* or provider* or professional* or personnel* or resident* or staff* or team or teams).kf. | #20 |
| Health Professionals | 19 or 20 | #21 |
| Primary Care (Controlled Vocabulary) | Primary Health Care/ OR General Practice/ or Family Practice/ or Physicians, Family/ or General Practitioners/ or Physicians, Primary Care/ or Group practice/ or Ambulatory Care/ OR Community Health Services/ OR Community Health Centers/ or Community Mental Health Services/ or Community Mental Health Centers/ OR Rural health services/ or Nurse practitioners/ or exp Preventive Health Services/ | #22 |
| Primary Care (Free text) | ((primary adj3 care) or ("primary healthcare" or "primary health" or "first line") or ((family or general or group) adj2 (doctor or doctors or physician* or practice* or medicine or nurs*)) or (rural adj3 (physician* or practice or service*)) or generalist* or (ambulatory adj2 (care or clinic)) or (health adj3 (center* or centre*)) or consult* or (visit* adj3 (clinic* or care or outpatient)) or (community adj3 (care or worker* or service* or nurs*)) or "clinical practice*" or (preventive* adj3 (care or cares or medicine* or service* or health*))).ti. or ((primary adj3 care) or ("primary healthcare" or "primary health" or "first line") or ((family or general or group) adj2 (doctor or doctors or physician* or practice* or medicine or nurs*)) or (rural adj3 (physician* or practice or service*)) or generalist* or (ambulatory adj2 (care or clinic)) or (health adj3 (center* or centre*)) or consult* or (visit* adj3 (clinic* or care or outpatient)) or (community adj3 (care or worker* or service* or nurs*)) or "clinical practice*" or (preventive* adj3 (care or cares or medicine* or service* or health*))).ab. or ((primary adj3 care) or ("primary healthcare" or "primary health" or "first line") or ((family or general or group) adj2 (doctor or doctors or physician* or practice* or medicine or nurs*)) or (rural adj3 (physician* or practice or service*)) or generalist* or (ambulatory adj2 (care or clinic)) or (health adj3 (center* or centre*)) or consult* or (visit* adj3 (clinic* or care or outpatient)) or (community adj3 (care or worker* or service* or nurs*)) or "clinical practice*" or (preventive* adj3 (care or cares or medicine* or service* or health*))).kf. | #23 |
| Primary Care | 22 or 23 | #24 |
| Total result | #15 AND #18 AND #21 AND #24 | #25 |
